# Factor Structure and Psychometric Properties of the Spence Children’s Anxiety Scale: A 25-Year Systematic Review

**DOI:** 10.1007/s10578-023-01566-1

**Published:** 2023-07-25

**Authors:** Teresa Galán-Luque, Marina Serrano-Ortiz, Mireia Orgilés

**Affiliations:** AITANA Research Group, Department of Health Psychology, Miguel Hernández University of Elche, Altamira Building. Ave. de la Universidad, 03202 Elche, Spain

**Keywords:** SCAS, Spence Children’s Anxiety Scale, Psychological assessment, Children and adolescents, Systematic review

## Abstract

The Spence Children’s Anxiety Scale (SCAS) is one of the most used instruments to assess anxiety symptoms in children and adolescents. Extensive research has been conducted to examine its psychometric properties and to develop other versions of the scale. The objective of this study was to examine the psychometric properties and factorial structure of the SCAS across different versions and populations. This systematic review followed PRISMA guidelines and was registered. APA PsycINFO, Web of Science (Core Collection) and MEDLINE (PubMed) were searched. Fifty-two studies were included in this systematic review. They examined the factor structure, convergent and divergent validity, and internal consistency of the scale. The most supported model was the original six-factor model, followed by the higher order six-factor model for the long version of the SCAS. Studies provided evidence of convergent validity and internal consistency. It is concluded that the SCAS is a valid and reliable instrument for assessing anxiety symptoms in children and adolescents, with a six-factor model structure well supported in most populations. Further research on the psychometric properties and factor structure of other versions of the scale and its application to clinical populations is warranted.

**Supplementary Information:**

The online version contains supplementary material available at 10.1007/s10578-023-01566-1.

## Introduction

In 2019, 58 million children and adolescents were living with an anxiety disorder (AD) worldwide [1]. ADs are characterized by the experience of impairing fear and worry and are related to behavioral problems [2]. In childhood, ADs are one of the most prevalent and impairing mental health problems and usually co-occur with other disorders, especially depression [3]. According to the World Health Organization [4], around 3.6% of children between the ages of 10–14 experience an AD. If not recognized and treated properly, ADs can become chronic, severely impact children and adolescents’ quality of life, and lead to subsequent adult negative psychosocial functioning [5]. Therefore, having valid and reliable self-report instruments becomes crucial to early detect anxiety symptoms [6].

The Spence Children’s Anxiety Scale (SCAS) [7, 8] is widely used by clinicians and researchers due to several reasons [6, 9]: (1) the SCAS was originally designed for children and adolescents, what makes it more specific for this population [7, 8]; (2) it comprises symptoms of the most prevalent DSM-5 ADs [2, 7, 8]; (3) the scale has shown good psychometric properties and its factor structure has been previously confirmed in several studies (e.g., [6, 10]); and (4) it is cost-efficient and provides sufficient clinical information to guide diagnosis and treatment efficiently [6, 11]. The SCAS was originally developed as a self-report measure that assessed the severity of anxiety symptoms in Australian children and adolescents from 8 to 14 years old [7]. More recently, there has been extensive research on studying the psychometric properties of the scale on samples from many different countries, and efforts have been made on the development of other versions of the scale, such as the parent reported [12], the teacher reported [13, 14], the preschoolers’ version (PAS) [15], and shortened versions of the scale [16].

Although there are many studies that have proven the SCAS as a valid and reliable assessment instrument and there is extensive literature supporting the six-factor model found in original studies in samples from different cultures [7, 8, 17], it should be noted that certain variability has been found in the psychometric properties of the scale [9, 18] and other models rather than the pioneer six-factor model have shown better fit for their data [18]. The SCAS is considered to be one of the most commonly used scales in both clinical practice and research [9], but systematic research efforts are needed to synthesize available and recent evidence on the factor structure and psychometric properties of the scale for several reasons. First, the data from the previously published systematic review on the psychometric properties of the scale [6] had to be updated as several studies were published afterwards [19, 20]. Second, this meta-analysis included only the self-reported version of the SCAS. In this regard, given the extensive use of the preschool, brief and parent versions of the scale, there was a need to summarize the available literature on the psychometric properties and factor structure of these versions.

Therefore, this study aimed at conducting a systematic review that synthesized the psychometric properties and factorial structure of the SCAS across different versions and populations. The specific objectives were: (1) to summarize and describe the available studies aimed at examining the psychometric properties and/or factor structure of the SCAS; (2) to determine the methods and number of factors that best fit the data from the different populations, and (3) to assess the convergent validity, divergent validity, and reliability of the different versions of the scale.

## Method

### Registration and Guidelines

The systematic review was performed according to an established protocol registered on PROSPERO (Registration Number: CRD42022365563). This study followed the Preferred Reporting Items for Systematic Reviews and Meta-analyses (PRISMA 2020) statement [21–23].

### Search Procedure and Eligibility Criteria

This systematic review examined the studies aimed at describing the psychometric properties of the SCAS in all its versions (i.e., self-report, parent, or teacher versions) in both community and clinical samples. A comprehensive search was performed by two authors (MSO and TGL) in the following bibliographic databases: APA PsycINFO, Web of Science (Core Collection) and MEDLINE (PubMed). The following terms were combined as follows: (“Spence Children Anxiety Scale” OR “SCAS” OR “Preschool Anxiety Scale”) AND (“psychometric properties” OR “factor analys*” OR “factor structure*” OR “validity” OR “validation”). The detailed search strategy can be found in Appendix 1.

The Population, Intervention, Comparator, Outcome, Study Design (PICOS) statement [24] was used to establish the following eligibility criteria:

- Population: children or adolescents under the age of 18 inclusive, and their parents or teachers for the parent and teacher versions, respectively. Community and clinical samples were included.
- Intervention or exposure: the SCAS [7] in all its versions.
- Comparison: other instruments that assess anxiety or other symptoms for the calculation of the convergent and divergent validity of the SCAS, respectively.
- Outcomes: the psychometric properties or the factor structure of the SCAS.

Studies were excluded if they did not report the psychometric properties or the factor structure of the SCAS, if the sample included adolescents over 18 years of age, or if they were not written in English or Spanish.

An Excel file was created to export all the results and duplicates were deleted. Two authors (MSO and TGL) independently screened the remaining records by title and abstract. They then independently screened the records by full-text. Disagreements were consulted to a third author (MOA) and agreement was reached by consensus.

### Data Extraction

Two authors (MSO and TGL) extracted the data independently using previously designed data extraction forms in an Excel file. For Table 1, the following data was extracted: first author and year of publication, version of the scale (PAS or SCAS, and if it was the brief version [yes/no]), informant (parent, child, or teacher), type of population (community or clinical), country of study, sample size, percentage of females (%), and age range. For Table 2, regarding the factor structure of the scale, the following information was extracted: mean age (and standard deviation), methods used (exploratory factor analysis [EFA] and/or confirmatory factor analysis [CFA]), number of factors, and percentage of variance explained. For Table 3, about the psychometric properties, it was extracted: Pearson’s *r* or Spearman’s *ρ* for convergent and divergent validity, and Cronbach’s alpha for the SCAS total score, subscales (Cronbach’s alpha range), and test–retest.

**Table 1:** Characteristics of the studies included in the systematic review

Reference	SCAS/PAS	Short version	Informant	Type of population	Country of study	Sample size (N)	Female (%)	Age range (min–max)
Broeren & Muris (2008)	PAS	No	P	C	The Netherlands	275	57.09	2–6
Wang & Zhao (2015)	PAS	No	P	C	China	1854	46.71	3–6
Leung et al. (2018)	PAS	No	P	C	China	1317	49.1	3–6
GuðmundsdÓttir et al. (2019)	PAS	No	P	C	Iceland	255	47	4–6
Maharjan et al. (2022)	PAS	No	P	C	Nepal	680	44.26	3–6
Edwards et al. (2010)	PAS	No	P	C/CL	Australia	764	50.3	3–5
Rodríguez-Menchón et al. (2022)	SCAS	Yes	CH	C	Spain	824	52.3	8–12
Deeba et al. (2015)	SCAS	Yes	CH	C/CL	Bangladesh	C = 583 CL = 777	65.29	9–17
Ahlen et al. (2018)	SCAS	Yes	CH	C/CL	Sweden	C1 = 750 C2 = 392 CL = 93	C = 49.5 CL = 55	C = 8–13 CL = 8–12
Orgilés et al. (2022)	SCAS	Yes	P	C	Spain	215	47.6	8–12
Gong et al. (2021)	SCAS	Yes	P/CH	C	China	CH = 478 P = 948	48.5	9–13
Reardon et al. (2018)	SCAS	Yes	P/CH/T	C/CL	England	C = 361 CL = 338	C = 53.18 CL = 50.29	7–11
Spence (1997) Study 1	SCAS	No	CH	C	Australia	698	60.89	8–12
Spence (1997) Study 2	SCAS	No	CH	C	Australia	698	59.45	8–12
Spence (1998)	SCAS	No	CH	C	Australia	584	59.99	9–12
Essau et al. (2002)	SCAS	No	CH	C	Germany	556	50.54	8–12
Muris et al. (2002)	SCAS	No	CH	C	South Africa	591	49.075	N/A
Muris et al. (2002)	SCAS	No	CH	C	Belgium	521	59.93	12–18
Spence et al. (2003)	SCAS	No	CH	C	Australia	875	46	13–14
Tortella-Feliu et al. (2005)	SCAS	No	CH	C	Spain	692		
Mellon & Moutavellis (2007)	SCAS	No	CH	C	Greece	1520	48.8	9–12
Essau et al. (2008) Study 1	SCAS	No	CH	C	China	428	51.9	12–17
Essau et al. (2008) Study 2	SCAS	No	CH	C	Germany	594	59.4	12–17
Ishikawa et al. (2009)	SCAS	No	CH	C	Japan	2225	49.48	9–15
Hernández-Guzmán et al. (2010)	SCAS	No	CH	C	Mexico	554	49.85	8–12
Essau et al. (2011)	SCAS	No	CH	C	Germany, Cyprus, England, Sweden & Italy	2558	58.40	12–17
Essau et al. (2011)	SCAS	No	CH	C	Cyprus	1072	57.7	12–17
Godoy et al. (2011)	SCAS	No	CH	C	Spain	1671	51.23	10–17
Carrillo et al. (2012)	SCAS	No	CH	C	Spain	1636	51	9–17
Essau et al. (2012)	SCAS	No	CH	C	Iran	1984	50.7	12–17
Orgilés et al. (2012)	SCAS	No	CH	C	Spain	1708	49.4	8–12
Zhao et al. (2012)	SCAS	No	CH	C	China	1878	49.31	8–15
Di Riso et al. (2013)	SCAS	No	CH	C	Italy	1397	49	8–10
Orgilés et al. (2013)	SCAS	No	CH	C	Spain	1374	52	13–17
Tsocheva et al. (2013)	SCAS	No	CH	C	Bulgaria	700	46.1	13–17
Ishikawa et al. (2018)	SCAS	No	CH	C	Japan	1500	50.6	15–18
Qadir et al. (2018)	SCAS	No	CH	C	Pakistan	1277	44.55	13–17
Forcadell et al. (2021)	SCAS	No	CH	CL	Spain	130	48.9	6–17
Ishikawa et al. (2014)	SCAS	No	P	C	Japan	677	CH = 50.37 P = 83.90	9–12
Orgilés et al. (2019)	SCAS	No	P	C	Spain	181	45.9	6–8
Nauta et al. (2004)	SCAS	No	P	C/CL	Australia & The Netherlands	C = 261 CL = 482	CL = 45 C = 52	CL = 6–17 C = 6–18
Li et al. (2016)	SCAS	No	P	C/CL	China & Italy	China = 456 Italy = 452	China = 59 Italy = 59.3	12–18
Zainal et al. (2014)	SCAS	No	P	CL	Singapore	32	N/A	6–18
Glod et al. (2017)	SCAS	No	P	CL	England	ASD = 285 ADs = 224	ASD = 13.68 ADs = 33.04	8–17
Jitlina et al. (2017)	SCAS	No	P	CL	Canada	238	16.4	8–11
Magiati et al. (2017)	SCAS	No	P	CL	England, Singapore & United States	870	12.3	5–18
Li et al. (2011)	SCAS	No	P/CH	C	China	207	50.24	6–11
DeSousa et al. (2014)	SCAS	No	P/CH	C	Brazil	712	53.1	7–17
Ahmadi et al. (2015)	SCAS	No	P/CH	C	Malaysia	CH = 600 P = 424	49.7	9–11
Whiteside & Brown (2008)	SCAS	No	P/CH	C/CL	United States	C = 85 CL = 85	N/A	9–18
Arent et al. (2014)	SCAS	No	P/CH	C/CL	Denmark	C CH = 972 C P = 805 CL CH = 268	N/A	7–17
Wang et al. (2016)	SCAS	No	P/CH	C/CL	China	C CH = 1785 C P = 1943 CL CH = 87 CL P = 77	C P = 47.86 CL CH = 58.44	7–15
Olofsdotter et al. (2016)	SCAS	No	P/CH	C/CL	Sweden	104	59.6	12–18
Carruthers et al. (2020)	SCAS	No	P/CH	CL	England	49	0	10–16

*PAS* Preschool Anxiety Scale; *SCAS* Spence Children’s Anxiety Scale; *P* Parent; *CH* Child; *T* Teacher; *C* Community; *CL* Clinical; *N/A* Not Available; *ADs* Anxiety Disorders; *ASD* Autism Spectrum Disorders

**Table 2:** Factor structure of SCAS derived from the studies included in the systematic review

Reference	Age *M (SD)*	Method	Number of factors	Explained variance (*%*)
Broeren & Muris (2008)	4.42 (1.07)	EFA	5	48.91
Wang & Zhao (2015)	4.93 (0.95)	CFA	5	N/A
Leung et al. (2018)	N/A	CFA	5	N/A
GuðmundsdÓttir et al. (2019)	N/A	EFA / CFA	4	50.3
Maharjan et al. (2022)	4.82 (N/A)	CFA	5	N/A
Edwards et al. (2010)	3.94 (0.53)	CFA	4^a^	N/A
Gong et al. (2021)	10.45 (0.85)	CFA	5	N/A
Rodríguez-Menchón et al. (2022)	9.64 (1.20)	CFA	1	N/A
Deeba et al. (2015)	12.3 (2.12)	CFA	1	N/A
Ahlen et al. (2018)	10.06 (N/A)	EFA	5	53.7%
Orgilés et al. (2022)	9.73 (1.23)	CFA	1	N/A
Reardon et al. (2018)	C = 9.50 (1.09) / CL = 9.70 (1.36)	N/A	N/A	N/A
Spence (1997)—Study 1	10.19 (1.3)	CFA	6^a^^,b^	N/A
Spence (1997)—Study 2	10.6 (1.31)	CFA	6^a^^,b^	N/A
Spence (1998)	10.32 (1.12)	CFA / EFA	CFA: 6^a,b^ / EFA: 6^c^	47
Essau et al. (2002)	10.6 (1.2)	EFA	5	43.8
Muris et al. (2002)	N/A	EFA	4	38.5
Muris et al. (2002)	15.1 (2)	N/A	N/A	N/A
Tortella-Feliu et al. (2003)	13.51 (0.51)	CFA / EFA	CFA: 6^a,b^ / EFA: 6^c^	47
Servera et al. (2005)	13.34 (1.52)	PCA	6	40.25%
Mellon & Moutavellis (2007)	N/A	EFA	6^c^	42%
Essau et al. (2008)—Study 1	13.8 (1.0)	CFA / EFA	5	N/A
Essau et al. (2008)—Study 2	14.6 (1.6)	CFA / EFA	6	N/A
Ishikawa et al. (2009)	N/A	CFA	5/6^a,b^	N/A
Hernández-Guzmán et al. (2010)	9.54 (1.34)	CFA	6^b^	N/A
Essau et al. (2011)	14.56 (1.6)	CFA	6^a^	N/A
Essau et al. (2011)	14.78 (1.7)	CFA	6^a^	N/A
Godoy et al. (2011)	13.21 (1.82)	CFA	6^a^^,b^	N/A
Carrillo et al. (2012)	13.26 (1.87)	N/A	N/A	N/A
Essau et al. (2012)	14.49 (1.7)	CFA	6^6^	N/A
Orgilés et al. (2012)	9.43 (1.15)	CFA	6^a^	N/A
Zhao et al. (2012)	12.42 (1.79)	CFA	6^a^	N/A
Di Riso et al. (2013)	9.04 (0.78)	CFA	6^a^	N/A
Orgilés et al. (2013)	14.3 (1.22)	CFA	6^a^	N/A
Tsocheva et al. (2013)	15.31 (1.00)	CFA	6^a^	N/A
Ishikawa et al. (2018)	12.01 (1.81)	CFA / EFA	CFA: 6^b^ / EFA: 5	N/A
Qadir et al. (2018)	N/A	CFA	6	N/A
Forcadell et al. (2021)	11.68 (2.68)	CFA	6^a^	N/A
Ishikawa et al. (2014)	N/A	CFA	5 / 6^a,b^	N/A
Orgilés et al. (2019)	6.87 (0.78)	CFA	6^a^	N/A
Nauta et al. (2004)	10.8 (2.4)	CFA	6^a^^,c^	53.4%
Li et al. (2016)	14.24 (1.90)	CFA	6^a^	N/A
Zainal et al. (2014)	10.3 (N/A)	N/A	N/A	N/A
Glod et al. (2017)	ASD = 12.33 (2) / ADs = 12.08 (2.74)	CFA / EFA	EFA: 6 (ASD) / 7 (ADs)	N/A
Jitlina et al. (2017)	8.9 (1.1)	CFA	*	N/A
Magiati et al. (2017)	11.6 (2.77)	CFA / PCA	CFA* / PCA = 5	N/A
Li et al. (2011)	N/A	CFA	6^a^ / 5	N/A
DeSousa et al. (2014)	11.52 (2.11)	CFA	6^a^	N/A
Ahmadi et al. (2015)	10.17 (0.77)	CFA	5	N/A
Whiteside & Brown (2008)	12.97 (2.59)	N/A	N/A	N/A
Arent et al. (2014)	C = 11.42 (2.36) / CL = 11.44 (2.16)	CFA	6^a^	N/A
Wang et al. (2016)	N/A	CFA	6^a^	N/A
Olofsdotter et al. (2016)	15.8 (1.5)	N/A	N/A	N/A
Carruthers et al. (2020)	12.88 (1.92)	N/A	N/A	N/A

*PAS* Preschool Anxiety Scale; *SCAS* Spence Children’s Anxiety Scale; *P* Parent; *CH* Child; *T* Teacher; *C* Community; *CL* Clinical; *N/A* Not Available; *ADs* Anxiety Disorders; *ASD* Autism Spectrum Disorders; *ECA* Exploratory Factor Analysis; *CFA* Confirmatory Factor Analysis; *PCA* Principal Component Analysis

^a^The study examined and found support for the original six-correlated factor model (Spence, 1997, 1998; Spence et al., 2003)

^b^The study examined and found support for the original six-factor, higher-order model proposed (Spence, 1997, 1998; Spence et al., 2003)

^c^Six-factor solution corresponding to the SCAS subscales (Spence, 1997, 1998; Spence et al., 2003)

^*^No models provided a good fit for the data

**Table 3:** Psychometric properties of the SCAS derived from the studies included in the systematic review

References	Convergent validity (Pearson’s *r*/Spearman’s *ρ*)	Divergent validity (Pearson’s *r* / Spearman’s *ρ*)	Reliability (Cronbach’s *α*)	Subscales’ Reliability (Cronbach’s *α*)	Test–retest reliability (Cronbach’s *α*)
Broeren & Muris (2008)	.77 (CMFWQ)	N/A	.86	.59–.81 (SAD–SOP)	N/A
Wang & Zhao (2015)	.31–.59 (CBCL-Int)	.21–.40 (CBCL-Ext)	.87	.55–.75 (SAD–SOP)	.73
Leung et al. (2018)	.497 (SDQ-Int)	.258 (SDQ-Ext)	.90	.64–.77 (SAD–SOP)	N/A
GuðmundsdÓttir et al. (2019)	.686 (SDQ-Emot)	.151 (SDQ-CPr) .023 (SDQ-HyIn)	.908	.725–.853 (SP–SOP)	N/A
Maharjan et al. (2022)	N/A	N/A	.87	.56–.75 (OCD–PIF)	N/A
Edwards et al. (2010)	Mo = .70 (SDQ-Emot) Fa = .62 (SDQ-Emot)	Mo = .14 (SDQ-CPr) Fa = .15 (SDQ-CPr) Mo = .07 (SDQ-Hy) Fa = .04 (SDQ-Hy)	Mo = .92 Fa = .92	Mo = .72–.89 (SP–SOP) Fa = .74–.89 (SP–SOP)	CH = .76 ADO = .86
Gong et al. (2021)	.28 (CPIC-CP) .16 (ERQ-Sup)	−.21 (CHS)	CH = .82 P = .89	CH = .61–.83 (SAD–PA) P = .67–.83 (SAD–PA)	N/A
Rodríguez-Menchón et al. (2022)	.43 (SDQ-Emot)	−0.01–-0.08 (SDQ-CPr) .02–.09 (SDQ-Hy)	.75	N/A	.77
Deeba et al. (2015)	.60 (CRIES-13)	N/A	.84	N/A	.80
Ahlen et al. (2018)	.95 (SCAS-S) .46 (RSE) .41 (ADIS) .53 (SCAS-P) .34 (SDQ-Emot)	.08 (SDQ-CPr)	.88	.68–.77 (PIF–GAD)	N/A
Orgilés et al. (2022)	.65 (SDQ-Emot) .36 (SDQ-PePr) .32 (SDQ-Hy) .62 (SDQ-Int) .30 (SDQ-Ext) .53 (SDQ)	.18 (SDQ-CPr) .00 (SDQ-Pros)	.82	N/A	N/A
Reardon et al. (2018)	.62–.76 (SDQ-Emot) .58-.70 (SDQ-Int)	.08–.32 (SDQ-CPr) .10–.34 (SDQ-Ext)	N/A	C = .84–.84 CL = .73–.85	N/A
Spence (1997)—Study 1	N/A	N/A	N/A	N/A	N/A
Spence (1997)—Study 2	N/A	N/A	N/A	N/A	N/A
Spence (1998)	.71 (RCMAS)	.48 (CDI)	.92	.60–.82 (PIF–PA)	.60
Essau et al. (2002)	.85 (SCARED) .71 (RCMAS) .41 (CIS) .67 (YSR)	N/A	.92	.57–.82 (PIF–PA)	.60
Muris et al. (2002)	.76 (SCARED)	N/A	.92	N/A	N/A
Muris et al. (2002)	.71 (MASC) .84 (SCARED) .79 (STAIC) .76 (RCMAS) .76 (FSSC-R)	.72 (CDI)	.93	.54–.83 (PIF–PA)	N/A
Spence et al. (2003)	.75 (RCMAS)	.60 (CDI)	.92	.60–.80 (PIF–PA)	.63
Tortella-Feliu et al. (2005)	.71 (CASI) .72 (STAIC-R)	.46 (CDI)	.87	.42–.75 (SP–PA)	.74
Mellon & Moutavellis (2007)	−.19 (TRF)	N/A	.90	.56–.78 (OCD–PA)	.83
Essau et al. (2008)—Study 1	N/A	N/A	N/A	N/A	N/A
Essau et al. (2008)—Study 2	N/A	N/A	N/A	N/A	N/A
Ishikawa et al. (2009)	CH = .47 (DSRS) ADO = .51 (DSRS)	N/A	CH = .94 ADO = .92	CH = .61–.85 (PIF–PA) ADO = .60–.79 (PIF–PA)	CH = .76 ADO = .86
Hernández-Guzmán et al. (2010)	.70 (ITA-UNAM)	.56 (CES-D)	.88	.71–.81 (SOP–PA)	N/A
Essau et al. (2011)	Ge = .59 (SDQ-Emot) Cy = .46 (SDQ-Emot) Eng = .74 (SDQ-Emot) Sw = .49 (SDQ-Emot) It = .71 (SDQ-Emot)	Ge = .17/.08 (SDQ-CPr/Hy) Cy = .13/.19 (SDQ-CPr/Hy) Eng = .33/.60 (SDQ-CPr/Hy) Sw = .23/.04 (SDQ-CPr/Hy) It = -.03/.03 (SDQ-CPr/Hy)	Ge = .89 Cy = .91 Eng = .97 Sw = .93 It = .91	Ge = .58–.75 (OCD–PA) Cy = .65–.80 (OCD–PA) Eng = .70–.90 (OCD–PIF) Sw = .71–.77 (SAD&SOP–GAD) It = .61–.79 (SAD–PA)	N/A
Essau et al. (2011)	.40 (CIS) .50 (SDQ) .44 (YSR) .53 (YSR-Int)	.16 (YSR-Ext)	.92	.61–.77 (PIF–PA)	.88
Godoy et al. (2011)	N/A	N/A	N/A	N/A	N/A
Carrillo et al. (2012)	.63 (BAI) .52 (CY-BOCS) .60 (WAQ) .57 (SDQ)	.31 (CDI-S) .69 (SDQ-CPr) .23 (SDQ-Hy) .31 (SDQ-PePr) .19 (SDQ-Pros)	.92	.61–.81 (PIF&SAD–PA)	.61
Essau et al. (2012)	.56 (SDQ)	.49 (CES-DC)	.92	.65–.83 (PIF–PA)	.61
Orgilés et al. (2012)	.41 (STAIC) −.65 (CHIP-CE)	−.004 (CDI)	.89	.52–.76 (N/A–N/A)	N/A
Zhao et al. (2012)	.82 (SCARED)	.53 (CDI)	.92	.64–.80 (PIF–PA)	.78
Di Riso et al. (213)	.41 (SDQ) .55 (SDQ-Int)	.13 (SDQ-CPr) .14 (SDQ-Hy) .18 (SDQ-Ext)	.91	.50–.76 (PIF–PA)	N/A
Orgilés et al. (2013)	.64 (SAS-A) .63 (STAIC)	.23 (RADS)	.89	.52–.75 (PIF–PA)	N/A
Tsocheva et al. (2013)	.51 (SDQ) .52 (CES-D)	.18 (SDQ-HyIn)	.92	.63–.82 (PIF–SAD)	N/A
Ishikawa et al. (2018)	.55 (DSRS)	N/A	.92	.52–.89 (PIF–SOP)	.76
Qadir et al. (2018)	.42 (SDQ-Emot)	.00 (SDQ-Pros)	.87	.53- .70 (SAD–PA)	N/A
Forcadell et al. (2021)	.51 (CBCL-ADs) .51 (CBCL-Int)	.43 (CBCL-Affec) .34 (CBCL-Ext)	.83	.49–.83 (PIF–PA)	.91
Ishikawa et al. (2014)	.51 (CBCL)	N/A	.88	.58–.75 (GAD–PA)	N/A
Orgilés et al. (2019)	.52 (MFQ) .53 (CALIS-P) .60 (SDQ-Int)	.21 (SDQ) −.27 (SDQ-Pros)	.91	.58–.81	.79
Nauta et al. (2004)	C = .59 (CBCL-Int) CL = .55 (CBCL-Int)	C = .34 CL = .33 (CBCL-Ext)	.89	C = .58–.74 (PIF–SAD&SOP&OCD) CL = .61–.81 (PIF–PA)	N/A
Li et al. (2016)	Chi = .572 It = .503	Chi = . 303 It = . 227	Chi = .92–.94 It = .86–.87	Chi = .61–.87 (PIF–PA) It = .52–.77 (PIF–PA)	N/A
Zainal et al. (2014)	.48 (K-SADS) .65 (DBC-P)	N/A	.88	.60–.78 (SAD–PA)	N/A
Glod et al. (2017)	N/A	N/A	N/A	N/A	N/A
Jitlina et al. (2017)	.41–.57 (CBCL)	.21–.41 (CBCL-Ag) .19–.34 (CBCL-Ext) .14–.29 (CBCL-Del)	.65	.75–.81 (AGO–GAD)	N/A
Magiati et al. (2017)	.64 (DBC-Anx)	.47 (DBC-Disr)	.93	.55–.84 (PIF–PA)	N/A
Li et al. (2011)	CH = .74 (NASSQ) P = .35 (NASSQ)	N/A	N/A	.63–.88 (PIF–PA)	N/A
DeSousa et al. (2014)	CH = .81 (SCARED) CH = .53 (SDQ-Emot) P = .85 (SCARED-P)	.34 (SDQ-HyIn) .14 (SDQ-CPr)	.885	.587–.811 (PIF–PA)	.81
Ahmadi et al. (2016)	.53 (SCAS-P)	N/A	.86	.50–.63 (GAD–SAD)	N/A
Whiteside & Brown (2008)	.68 (SCAS & SCAS-P) CH =−.30/.65/.65 (AFARS-Pos/Neg/Phy) P =−.21/.43/.37 (AFARS-Pos/Neg/Phy)	N/A	CH = .94 P = .93	CH = .53–.84 (PIF–PA&OCD) P = .43–.84 (PIF–OCD)	N/A
Arent et al. (2014)	.73 (BYI-A) .58 (BYI-D) .50 (SDQ-Int)	.14 (SDQ-Ext)	CH C = .92 CH CL = .89 P C = .90 P CL = .87	CH C = .59–.80 (PIF–PA) CH CL = .48–.79 (PIF–PA) P C = .50–.77 (PIF&SOP–OCD) P CL = .51–.82 (PIF–PA)	CH = .84 P = .83
Wang et al. (2016)	.58 (CBCL-Int)	.42 (CBLC-Ext)	.90–.91	.63–.77 (PIF–PA)	Fa = .66 Mo = .72
Olofsdotter et al. (2016)	.74 (SCAS-P) .63 (K-SADS)	N/A	CH = .94 P = .91	CH = .65–.86 (PIF–PA) P = .56–.83 (PIF–SOP&GAD)	N/A
Carruthers et al. (2020)	N/A	N/A	CH = .93 P = .94	CH = .72–.82 P = .62–.83	N/A

*CMFWQ* Children's Moods Fears and Worries Questionnaire; *CBCL-Int* Children Behavior Checklist Internalizing Subscale; *CBCL-Ext* Children Behavior Checklist Externalizing Subscale; *SDQ-Ext* Strengths and Difficulties Questionnaires Externalizing Subscale; *SDQ-Int* Strengths and Difficulties Questionnaires Internalizing Subscale; *SDQ-Emot* Strengths and Difficulties Questionnaires Emotional Symptoms Subscale; *SDQ-CPr* Strengths and Difficulties Questionnaires Conduct Problems Subscale; *SDQ-HyIn* Strengths and Difficulties Questionnaires Hyperactivity-Inattention Subscales; *SDQ-Hy* Strengths and Difficulties Questionnaires Hyperactivity Subscale; *CRIES-13* Children's Revised Impact of Events Scale-13; *SCAS-S* Spence Children’s Anxiety Scale—Short Version; *CSR* Clinician Severity Ratings in ADIS; *ADIS* Anxiety Disorders Interview Schedule; *SCAS-P* The Spence Children’s Anxiety Scale—Parent version; *SDQ-Pe* Strengths and Difficulties Questionnaires Peer Problems Subscale; *SDQ* Strengths and Difficulties Questionnaires Total Score; *SDQ-Pros* Strengths and Difficulties Questionnaires Prosocial Behavior Subscale; *CPIC-CP* Children’s Perception of Interparental Conflict Scale Conflict Properties Subscale; *ERQ-Sup* Emotion Regulation Questionnaire Suppression Subfactor; *CHS* Children’s Hope Scale; *RCMAS* Revised Children's Manifest Anxiety Scale; *CDI-S* Children's Depression Inventory Short Version; *SCARED* Screen for Child Anxiety Related Emotional Disorders; *CIS* Columbia Impairment Scale; *YSR* Youth Self-Report; *MASC* Multidimensional Anxiety Scale for Children; *STAIC* State–Trait Anxiety Inventory for Children; *FSSC-R* Fear Survey Schedule for Children – Revised; *CASI* Childhood Anxiety Sensitivity Index; *STAIC-R* State–Trait Anxiety Inventory for Children—Revised; *TRF* Teacher’s Report Form; *ITA-UNAM* Inventario de Trastornos de Ansiedad; *CES-D* Escala de Depresión del Centro de Estudios Epidemiológicos; *YSR-Int* Youth Self-Report Internalizing Subscale; *YSR-Ext* Youth Self-Report Externalizing Subscale; *BAI* Beck Anxiety Inventory; *CY-BOCS* Children’s Yale-Brown Obsessive Compulsive Scale; *WAQ* Worry and Anxiety Questionnaire; *CES-DC* Centre for Epidemiological Studies Depression Scale for Children; *CHIP-CE* Child Health and Illness Profile-Children Edition; *CDI* Children Depression Inventory; *SAS-A* The Social Anxiety Scale for Adolescents; *RADS* Reynolds Adolescent Depression Scale; *DSRS* Depression Self-Rating Scale; *CBCL-Ads* Child Behavior Checklist Subscales for Anxiety Disorder; *CBCL-Affect* Child Behavior Checklist Subscales for Affective Disorders; *CBCL* Child Behavior Checklist; *MFQ* Mood and Feelings Questionnaire; *CALIS-P* Children’s Anxiety Life Interference Scale—Parent report; *K-SADS* Kiddie-Schedule for Schizophrenia and Affective Disorders; *DBC-P* Development Behavior Checklist-Parent Version; *CBCL-Ag* Child Behavior Checklist Aggressiveness Subscale; *CBCL-Del* Child Behavior Checklist Delinquency Subscale; *DBC-Anx* Development Behavior Checklist Anxiety Subscale; *DBC-Disr* Development Behavior Checklist Disruptive/Antisocial Subscale; *SCARED-P* Screen for Child Anxiety Related Emotional Disorders—Parents Version; *SCAS* Spence Children’s Anxiety Scale; *AFARS-Pos* Affect and Arousal Scale Positive; *AFARS-Neg* Affect and Arousal Scale Negative; *AFARS-Phy* Affect and Arousal Scale Physiological Arousal; *BYI-A* Beck Youth Inventories Scales for Anxiety; *BYI-D* Beck Youth Inventories Scales for Depression. *Ge* Germany; *Cy* Cyprus; *Eng* England; *Sw* Sweden; *It* Italy; *Chi* China. *CH* Children; *ADO* Adolescents; *P* Parents; *C* Community; *CL* Clinical. *Mo* mothers; *Fa* Fathers

## Results

### Identification of Articles

**Fig. 1:**
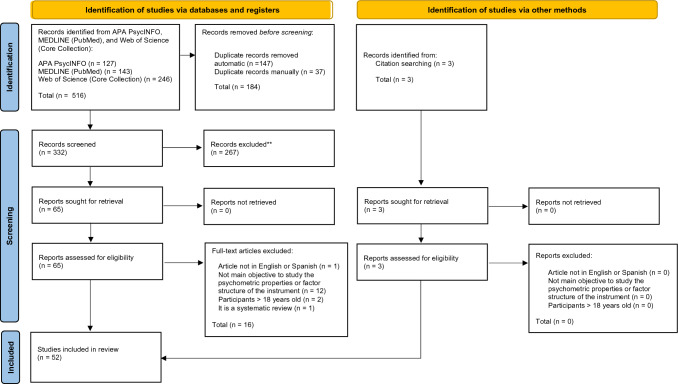
PRISMA Flow Diagram. *Note.* From “The PRISMA 2020 statement: an updated guideline for reporting systematic reviews”, by M. J. Page et al., 2021, BMJ, 372(71), p. 5 (https://doi.org/10.1136/bmj.n71). Distributed under the terms of the Creative Commons Attribution License

Figure 1 shows the PRISMA flow diagram for the literature search process. The initial search across all databases identified 516 records (332 records after removing duplicates). Three additional records were identified through citation searching. After screening by title and abstract, sixty-five records were full-text reviewed for eligibility and sixteen studies were excluded (reasons for exclusion for each record are presented in Appendix 2). Fifty-two studies were included in this systematic review.

### Studies and Sample Characteristics

The characteristics of the fifty-two included studies are shown in Table 1. For ease of reading, tables are organized systematically according to the version, informant, and type of population included in the study. The total sample consisted of 52,785 participants: 5,145 parents for the PAS; 4,596 children, 1,647 parents, and 215 teachers for the brief-version of the SCAS; and 34,375 children and 7,844 parents for the traditional version of the SCAS. Participants were from twenty-six different countries, the percentage of females ranged from 0% in the study of Carruthers et al. [25] to 83.90% in the study of Ishikawa et al. [26]; and the ages ranged from 2 to 6 years old for the PAS, and from 5 to 18 years old for the SCAS.

Since the publication of the original scale by Spence [7], eighteen articles have been published before 2012 and thirty-three in the last ten years. Of the twenty-two records, six focused on the preschool version of the SCAS, the PAS [20, 27–31]. Six reported data on the short version: four with the child as informant [16, 32–34]; one with the parent [35]; and one with the child, parent, and teacher [14]. The rest of the studies used the long version of the SCAS: twenty-four used the child-reported version, eight the parent-reported version, and eight used both.

Only six studies reported data exclusively on clinical population: children with any AD [19], children with autism spectrum disorders (ASDs) [25, 36–38], and children who presented both disorders [39].

### PAS and SCAS Factor Structure

Data regarding the PAS and SCAS factor structure is shown in Table 2. Of the six studies that examined the factor structure of the preschoolers’ version of the SCAS, only one study used exploratory factor analysis (EFA), four used confirmatory factor analysis (CFA), and one used both. Four studies supported a five-correlated-factor structure (i.e., social phobia [SOP], separation anxiety [SAD], generalized anxiety [GAD], obsessive–compulsive disorder [OCD] and physical injury fears [PIF]). The study of Maharjan et al. [20] found a better fit for the data after removing three items from the scale, i.e., item 2, 3, and 22. Two studies supported a four-correlated model (i.e., GAD, SOP, SA, and specific fears). The study by Edwards et al. [30] proposed all factors loading on a higher order “anxiety” factor, while GuðmundsdÓttir et al. [27] found a decent fit for a four-factor model in the EFA but not in CFA testing the four-factor model proposed by Edwards et al. [30].

Of the six studies focusing on the brief version of the SCAS (SCAS-S), only five reported data on the factor structure. Two studies validated the five-factor structure of the SCAS-S [16, 33]. The results of the factor analysis performed by Ahlen and colleagues found good support for a structure comprising five group factors loading upon a high general factor [16]. The rest of the studies revealed a good fit for the one-factor structure of the SCAS-S [32, 34, 35].

Thirty-five studies examined the SCAS factor structure both children and parent versions. Three studies used EFA, twenty-five used CFA, six used both EFA and CFA, one used Principal Component Analysis (PCA), and one used both CFA and PCA. Regarding the nine studies that used EFA, two of them were the original by Spence [8, 17], that found support for the six-factor model. Another study found support for this model [40], while the remaining six found support for a six-factor model but different from the original [39, 41], for a seven-factor model [39], for a five-factor model [10, 41, 42], and for a four-factor model [43]. About the studies using CFA, twenty-one found support for the original six-factor model [7, 8, 17], six for the original six-factor higher-order model [7, 8, 17], five for both. Four studies found a better fit for a five-factor model [26, 42, 44, 45].

### SCAS Psychometric Properties

Data regarding the psychometric properties of the SCAS is presented in Table 3. Several instruments have been used to test the validity of the scale, among them, it is to note the Strengths and Difficulties Questionnaire (SDQ) [46] or the Child Behavior Checklist (CBLC) [47]. Studies report correlations from 0.41 to 0.57 when comparing the scores of the SCAS with the total scores of the SDQ, and from 0.34 to 0.76 when comparing them with the Emotional Symptoms Subscale of that instrument. With other instruments especially designed to evaluate anxiety symptoms, higher correlations are found, ranging from 0.76 to 0.85. Concerning the divergent validity of the scale, it should be highlighted that only thirty of the fifty-two studies reported data on this regard. The most used scale was SDQ, especially the Hyperactivity, Inattention, and Conduct Problems Subscales. Correlations with these subscales ranged from 0.00 to 0.39; although the study of Carrillo and colleagues [48] found a correlation with the Conduct Problems Subscale of 0.69. The CDI, also used to test the divergent validity of the SCAS in seven studies, showed correlations from -0.004 to 0.72, which is indicative that constructs measured by both instruments are related.

Forty-four studies explored the reliability of the total scale, and forty-two reported data on the reliability of each subscale. Cronbach’s alphas for the total score ranged from 0.65 to 0.97, and from 0.42 to 0.90 for the subscales. Thirty-two studies of the forty-two that reported data concerning the reliability of the subscales found that the PIF subscale had the lowest value (0.43 to 0.68). Thirty-five studies of the forty-two found that the Panic Attack and Agoraphobia (PA) subscale had the highest values (0.70 to 0.87). Only twenty-seven studies explored the test–retest reliability, and Cronbach’s alphas ranged from 0.60 to 0.91.

## Discussion

The present systematic review aimed to summarize the available literature on the Spence Children’s Anxiety Scale in all its versions. It is the first to bring together all studies on the factor structure and the psychometric properties of the SCAS, including preschool, brief, parent, child, and teacher versions of the instrument. The specific objectives were: (1) to describe the studies aimed at examining the psychometric properties and/or factor structure of the SCAS; (2) to determine the factor structure that was best supported in the literature; and (3) to assess the validity and reliability.

First, it is important to note that valid and reliable measures for assessing anxiety symptoms in infant populations have attracted considerable attention in the literature over the past few years, as can be inferred from the increasing number of publications on the subject over the past decade and the large number of people participating in the studies (more than 50,000 people were involved in the studies included in this systematic review). Most of the studies focused on the long traditional version of the SCAS, although recent efforts are pivoting towards evaluating the properties of other versions of the scale, such as the preschool and brief versions of the SCAS, or in clinical populations (e.g., autism disorders), necessary to meet the growing demand for the assessment of anxiety symptomatology in other developmental stages, in different contexts (e.g., schools—through teacher versions of the scale), and in children suffering from other health problems.

Second, the results of the studies indicated that there is a variability in the factor structure that is best supported for the PAS and for the short version of the SCAS, warranting further research in this regard. Regarding the SCAS, the most supported model was the original six-factor model, followed by the higher six-factor model [7, 8, 17], confirming data from the previous systematic review about the factor structure of the children version of the scale [6]. Participants in this study came from twenty-six different countries, suggesting that this scale is applicable across countries. However, studies with participants from countries such as China [45], Japan [26, 42], Malaysia [44], Germany [10, 41], or England [39], found support for other factor structures. Differences in the structure are unlikely to be due solely to social factors, as samples from countries with similar cultural values were indeed able to replicate the original models [6]. Authors have proposed factors such as personality traits, experimental designs, or statistics to explain these differences [6, 41, 49]. In this sense, the study by Glod et al. [39], for example, found differences in the factor structures of the scale for children with anxiety disorders and autism disorders. Comorbidities with other health problems are therefore suggested as another factor that should be further investigated as a potential variable influencing the factor structure differences across population groups.

Third, studies reported high correlations between the SCAS and other scales, such as the SDQ or the SCARED, providing evidence of convergent validity. Lower correlations were found between anxiety and other constructs derived from the SDQ (i.e., conduct problems, hyperactivity, or inattention). Only two studies [32, 50] reported null correlations between the SCAS and the prosocial subscale of the SDQ, making it difficult to draw definite conclusions about divergent validity. Reliability for the total scale ranged from good to excellent for the PAS, from acceptable to good for the brief version, and from acceptable to excellent for the long version of the scale. Only one study [37] reported a questionable reliability (Cronbach’s alpha = 0.65), maybe since the sample was quite diverse, including children with ASD and intellectual disability. Further research to evaluate the reliability of the scale in clinical populations is warranted. The reliability of the PIF subscale ranged from unacceptable to questionable, previously explained by the low number of items and the variability of the situations they describe [51, 52]. The PA subscale showed the highest reliability in more than eighty percent of the studies, ranging from good to acceptable. Most studies reported good to acceptable test–retest reliability, showing evidence of the scale’s good temporal stability for measuring anxiety symptoms.

Finally, it is of utmost research and clinical significance to conduct a comparative of the Spence Children's Anxiety Scale (SCAS) in relation to other pertinent and widely employed measures utilized for assessing anxiety symptoms in children and adolescents, namely the Screen for Child Anxiety Related Disorders (SCARED) [53], the Multidimensional Anxiety Scale for Children (MASC) [54], and the Youth Anxiety Measure for DSM-5 (YAM-5) [55]. The development of the SCAS, SCARED, and MASC emerged in response to the clinical and research demands following the release of the Diagnostic and Statistical Manual of Mental Disorders, Fourth Edition (DSM-IV) in 1994 [56]. Conversely, the YAM-5 represents a more recent scale aligned with the DSM-5, which introduced modifications to the classification of anxiety disorders by excluding certain disorders (e.g., obsessive–compulsive disorder) and incorporating others (e.g., selective mutism) [57, 58]. Regarding the MASC-2, limitations have been previously documented by other scholars [59]. These include its cost, which is computed per purchased form, its limited availability in languages beyond English, and its relatively smaller research foundation when compared to the SCAS and SCARED [59]. In contrast, both the SCAS and SCARED have undergone extensive translation, validation, and research-based examination, thus promoting their widespread adoption and facilitating cross-cultural utilization of empirically grounded instruments by clinicians and researchers across countries. Although a prior meta-analysis published in 2018 suggested that the SCAS possesses a more limited research base than the SCARED [59], the past five years have witnessed the publication of over ten studies exploring the psychometric and factor structure of the SCAS, thereby providing evidence for the validity of its factor structure and psychometric properties. This surge in interest within the scientific community towards the utilization of various SCAS versions, including the abbreviated and preschool adaptations. A notable advantage of the SCAS, in comparison to the SCARED, may lie in its shorter length, as the longest version of the SCAS comprises 44 items, whereas the SCARED encompasses a range of 38 to 71 items, depending on the variant [59]. Additionally, recent efforts have been dedicated to developing the SCAS for the assessment of anxiety symptoms in children under the age of 8, as research indicates that certain anxiety disorders exhibit an onset peak before this age (e.g., specific phobias or separation anxiety disorder) [60]. Regarding the YAM-5, multiple studies have demonstrated its reliability and validity in assessing DSM-5 anxiety disorder symptoms [55, 61]. Nevertheless, as a newly developed measure, further exploration with diverse international samples is warranted, given its capacity to shed light on new diagnostic categories within the evolving classification systems that may not be captured by older instruments.

### Strengths, Limitations and Future Directions

This study is limited by several facts. First, although the search was exhaustive and multiple databases were searched, some studies may have been excluded, which may have influenced the conclusions drawn from the synthesized results. Second, factorial invariance and risk of bias were not examined in this paper and should be prioritized in future studies. Third, drawing conclusions about the psychometric properties and factor structure of the preschoolers’ and the short version of the scale and in clinical populations was limited by the sparse literature that has been published to date. We suggest this should be examined in further studies.

Despite these limitations, there are several notable strengths of this work. This study is based on the PRISMA guidelines, and all decisions made in the course of its development were reported, which contributes to transparency and makes it replicable to update the data in the future. In the present work, we extended our previous systematic review of the SCAS [6] by incorporating other versions of the scale along with some psychometric properties not previously explored by this meta-analysis (i.e., test–retest reliability). This research is hoping to contribute to the direction of helping clarify the fact that this scale, together with the obvious advantages in terms of its usefulness, has enough psychometric quality to be used in both clinical and research settings.

### Summary

This systematic review provides an overview of the studies that have examined the psychometric quality of one of the most widely used scales for assessing anxiety symptoms in children and adolescents, the Spence Children’s Anxiety Scale. This work followed PRISMA guidelines and included fifty-two studies exploring the psychometric properties or the factor structure of the scale. Most studies focused on the long version of the scale. Overall, it can be concluded that this version is a valid and reliable instrument for assessing anxiety symptoms in children and adolescents, with a six-factor model structure that is well supported in most populations. Further research on the psychometric properties and factor structure of other versions of the scale and its application to clinical populations is warranted. This systematic review expands the available knowledge on the SCAS, and in particular on the previously reported systematic review of the instrument, by including other versions of the scale and populations in their samples.

## Supplementary Information

Below is the link to the electronic supplementary material.

Supplementary material 1 (DOCX 27 kb)

## Data Availability

There are no other datasets associated with this systematic review beyond those presented in the manuscript and its appendix.
