# Supplementary material for: Factor Structure and Psychometric Properties of the Spence Children’s Anxiety Scale: A 25-Year Systematic Review
Source: Child Psychiatry Hum Dev. 2023 Jul 25;56(2):492–506. doi: 10.1007/s10578-023-01566-1 (PMC11928368; doi:10.1007/s10578-023-01566-1)
Supplement: Supplementary file 1 — Supplementary material 1 (DOCX 27 kb) [file 10578_2023_1566_MOESM1_ESM.docx]

**Appendix 1. Search Strategies**

**A.1. Detailed Search Strategy for MEDLINE (PubMed)**

· **Source:** Medline (PubMed)

· **Database coverage dates:** 1960 – 1965 (OLDMEDLINE) & 1966 – 2022 (Medline)

· **Search date:** 31/11/2022

· **Retrieved records:** 143

· **Search strategy:**

(("Spence Children's Anxiety Scale"[Title/Abstract] OR "scas*"[Title/Abstract] OR "Preschool Anxiety Scale"[Title/Abstract]) NOT ("sex chromosome"[Title/Abstract] OR "spinocerebellar ataxia*"[Title/Abstract] OR "spino cerebellar ataxia*"[Title/Abstract])) AND ("Psychometrics"[MeSH Terms] OR "factor analysis, statistical"[MeSH Terms] OR "Sensitivity and Specificity"[MeSH Terms] OR "Mass Screening"[MeSH Terms] OR "psychometric*"[Title/Abstract] OR "factor analys*"[Title/Abstract] OR "factor structure*"[Title/Abstract] OR "validity"[Title/Abstract] OR "validation"[Title/Abstract] OR "reliability"[Title/Abstract])

**A.2. Detailed Search Strategy for APA PsycINFO**

**· Source:** EBSCOhost

**· Database coverage dates:** 1806 – 2022

**· Search date:** 31/11/2022

**· Retrieved records:** 127

**· Search strategy:**

| **Search ID#** | **Search Terms** | **Limiters/Expanders** | **Results** |
| --- | --- | --- | --- |
| S13 | S4 AND S12 | **Expanders-** Apply equivalent materials  **Search modes** -Boolean/Phrase | 127 |
| S12 | S10 NOT S11 | **Expanders-** Apply equivalent materials  **Search modes** -Boolean/Phrase | 461 |
| S11 | S8 OR S9 | **Expanders-** Apply equivalent materials  **Search modes** -Boolean/Phrase | 1,845 |
| S10 | S5 OR S6 OR S7 | **Expanders-** Apply equivalent materials  **Search modes** -Boolean/Phrase | 533 |
| S9 | TI "cerebellar ataxia*" OR AB "cerebellar ataxia*" OR KW "cerebellar ataxia*" | **Expanders-** Apply equivalent materials  **Search modes** -Boolean/Phrase | 1,445 |
| S8 | TI "sex chromosome" OR AB "sex chromosome" OR KW "sex chromosome" | **Expanders-** Apply equivalent materials  **Search modes** -Boolean/Phrase | 400 |
| S7 | TI "Preschool Anxiety Scale" OR AB "Preschool Anxiety Scale" OR KW "Preschool Anxiety Scale" | **Expanders-** Apply equivalent materials  **Search modes** -Boolean/Phrase | 27 |
| S6 | TI "SCAS*" OR AB "SCAS*" OR KW "SCAS*" | **Expanders-** Apply equivalent materials  **Search modes** -Boolean/Phrase | 435 |
| S5 | TI "Spence Children's Anxiety Scale" OR AB "Spence Children's Anxiety Scale" OR KW "Spence Children's Anxiety Scale" | **Expanders-** Apply equivalent materials  **Search modes** -Boolean/Phrase | 187 |
| S4 | S1 OR S2 OR S3 | **Expanders-** Apply equivalent materials  **Search modes** -Boolean/Phrase | 398,826 |
| S3 | TI (factor structure or factor analys*) OR AB (factor structure or factor analys*) OR KW (factor structure or factor analys*) | **Expanders-** Apply equivalent materials  **Search modes** -Boolean/Phrase | 104,994 |
| S2 | TI (psychometric* or validity or reliability or psychometric properties ) OR AB (psychometric* or validity or reliability or psychometric properties ) OR KW (psychometric* or validity or reliability or psychometric properties ) | **Expanders-** Apply equivalent materials  **Search modes** -Boolean/Phrase | 248,685 |
| S1 | DE "Psychometrics" OR DE "Classical Test Theory" OR DE "Consistency (Measurement)" OR DE "Error of Measurement" OR DE "External Validity" OR DE "Factor Analysis" OR DE "Internal Validity" OR DE "Item Analysis (Test)" OR DE "Item Response Theory" OR DE "Measurement Invariance" OR DE "Measurement Models" OR DE "Multivariate Analysis" OR DE "Test Construction" OR DE "Test Reliability" OR DE "Test Sensitivity" OR DE "Test Specificity" OR DE "Test Validity" OR DE "Variability Measurement" | **Expanders-** Apply equivalent materials  **Search modes** -Boolean/Phrase | 229,761 |

**A.3. Detailed Search Strategy for Web of Science (Core Collection)**

**· Source:** Clarivate / FECYT

**· Database coverage dates:** 1945 – 2022

**· Search date:** 31/11/2022

**· Retrieved records:** 246

**· Search strategy:**

| **#** | **Query** | **Number of results** |
| --- | --- | --- |
| 1 | "Spence Children's Anxiety Scale" (Topic) | 215 |
| 2 | TS=("scas*") | 2,017 |
| 3 | TS=("Preschool Anxiety Scale") | 29 |
| 4 | TS=("sex chromosome*") | 11,630 |
| 5 | TS=("spinocerebellar ataxia*") | 7,086 |
| 6 | TS=("cerebellar ataxia*") | 8,055 |
| 7 | #1 OR #2 OR #3 | 2,133 |
| 8 | #4 OR #5 OR #6 | 24,826 |
| 9 | #7 NOT #8 | 1,553 |
| 10 | TS=("psychometric*") | 105,363 |
| 11 | TS=("factor analys*") | 122,156 |
| 12 | TS=("factor structure*") | 27,407 |
| 13 | TS=("validity") | 553,376 |
| 14 | TS=("validation") | 784,636 |
| 15 | TS=("reliab*") | 1,440,612 |
| 16 | #10 OR #11 OR #12 OR #13 OR #14 OR #15 | 2,601,639 |
| 17 | #9 AND #16 | 244 |
| 18 | #9 AND #16 and Spanish or English (Languages) | 242 |

**Appendix 2. Reasons for Exclusion**

| **Reason for exclusion** | **Studies** |
| --- | --- |
| Not a research article | Orgilés et al., 2016 |
| Article not in English or Spanish | Delvecchio et al., 2010 |
| Not main objective to study the psychometric properties or factor structure of the instrument | Ahmadi et al., 2015  Delvecchio et al., 2015  Desousa et al., 2012  Di Riso et al., 2014  Evans et al., 2017  Holly et al., 2015  Magiati et al., 2014  Myburgh et al., 2021  Reardon et al., 2019  Sattler et al., 2018  Whiteside et al., 2012 |
| Participants > 18 years old | Delvecchio et al., 2017  Muris et al., 2000 |

**References for Excluded Studies:**

Ahmadi, A., Mustaffa, M. S., Haghdoost, A., Khan, A., & Latif, A. A. (2015). Cross-cultural adaptation of the Spence Children’s Anxiety Scale in Malaysia. *Trends in Psychiatry and Psychotherapy*, *37*(1), 37–41. <https://doi.org/10.1590/2237-6089-2014-0038>

Delvecchio, E., Li, J.-B., Liberska, H., Lis, A., & Mazzeschi, C. (2017). The Polish Spence Children’s Anxiety Scale: Preliminary Evidence on Validity and Cross-Cultural Comparison. *Journal of Child and Family Studies*, *26*(6), 1554–1564. <https://doi.org/10.1007/s10826-017-0685-9>

Delvecchio, E., Mabilia, D., di Riso, D., Miconi, D., & Li, J.-B. (2015). A Comparison of Anxiety Symptoms in Community-Based Chinese and Italian Adolescents. *Journal of Child and Family Studies*, *24*(8), 2418–2431. <https://doi.org/10.1007/s10826-014-0045-y>

Delvecchio, E., di Riso, D., Chessa, D., & Lis, A. (2010). The Spence Children’s Anxiety Scale in Italian children aged 8-10. *Giunti Organizzazioni Speciali*, *261–262*, 117–123.

DeSousa, D. A., Petersen, C. S., Behs, R., Manfro, G. G., & Koller, S. H. (2012). Brazilian Portuguese version of the Spence Children’s Anxiety Scale (SCAS-Brasil). *Trends in Psychiatry and Psychotherapy*, *34*(3), 147–153. <https://doi.org/10.1590/S2237-60892012000300006>

Di Riso, D., Bobbio, A., Chessa, D., Lis, A., & Mazzeschi, C. (2014). Analysis of the interplay between depression, anxiety, and psychological resources in adolescence using self-report measures. *International Journal of Psychiatry in Clinical Practice*, *18*(2), 103–111. <https://doi.org/10.3109/13651501.2014.890227>

Evans, R., Thirlwall, K., Cooper, P., & Creswell, C. (2017). Using symptom and interference questionnaires to identify recovery among children with anxiety disorders. *Psychological Assessment*, *29*(7), 835–843. <https://doi.org/10.1037/pas0000375>

Holly, L. E., Little, M., Pina, A. A., & Caterino, L. C. (2015). Assessment of Anxiety Symptoms in School Children: A Cross-Sex and Ethnic Examination. *Journal of Abnormal Child Psychology*, *43*(2), 297–309. <https://doi.org/10.1007/s10802-014-9907-4>

Magiati, I., Chan, J. Y., Tan, W.-L. J., & Poon, K. K. (2014). Do non-referred young people with Autism Spectrum Disorders and their caregivers agree when reporting anxiety symptoms? A preliminary investigation using the Spence Children’s Anxiety Scale. *Research in Autism Spectrum Disorders*, *8*(5), 546–558. <https://doi.org/10.1016/j.rasd.2014.01.015>

Muris, P., Schmidt, H., & Merckelbach, H. (2000). Correlations among two self-report questionnaires for measuring DSM-defined anxiety disorder symptoms in children: the Screen for Child Anxiety Related Emotional Disorders and the Spence Children’s Anxiety Scale. *Personality and Individual Differences*, *28*(2), 333–346. <https://doi.org/10.1016/S0191-8869(99)00102-6>

Myburgh, N., Loxton, H., & Engels, R. C. M. E. (2021). Cross-cultural adaptation of an anxiety measure in a disadvantaged South African community context: Methodological processes and findings. *Transcultural Psychiatry*, *58*(6), 759–771. <https://doi.org/10.1177/13634615211011850>

Orgilés, M., Fernández-Martínez, I., Guillén-Riquelme, A., Espada, J. P., & Essau, C. A. (2016). A systematic review of the factor structure and reliability of the Spence Children’s Anxiety Scale. *Journal of Affective Disorders*, *190*, 333–340. <https://doi.org/10.1016/j.jad.2015.09.055>

Reardon, T., Creswell, C., Lester, K. J., Arendt, K., Blatter-Meunier, J., Bögels, S. M., Coleman, J. R. I., Cooper, P. J., Heiervang, E. R., Herren, C., Hogendoorn, S. M., Hudson, J. L., Keers, R., Lyneham, H. J., Marin, C. E., Nauta, M., Rapee, R. M., Roberts, S., Schneider, S., … Eley, T. C. (2019). The utility of the SCAS-C/P to detect specific anxiety disorders among clinically anxious children. *Psychological Assessment*, *31*(8), 1006–1018. <https://doi.org/10.1037/pas0000700>

Sattler, A. F., Whiteside, S. P. H., Bentley, J. P., & Young, J. (2018). Development and validation of a brief screening procedure for pediatric obsessive-compulsive disorder derived from the Spence Children’s Anxiety Scale. *Journal of Obsessive-Compulsive and Related Disorders*, *16*, 29–35. <https://doi.org/10.1016/j.jocrd.2017.12.004>

Whiteside, S. P. H., Gryczkowski, M. R., Biggs, B. K., Fagen, R., & Owusu, D. (2012). Validation of the Spence Children’s Anxiety Scale’s obsessive-compulsive subscale in a clinical and community sample. *Journal of Anxiety Disorders*, *26*(1), 111–116. <https://doi.org/10.1016/j.janxdis.2011.10.002>
